# Low Threshold Current and Polarization-Stabilized 795 nm Vertical-Cavity Surface-Emitting Lasers

**DOI:** 10.3390/nano13061120

**Published:** 2023-03-21

**Authors:** Qiuxue Fu, Yurun Sun, Suzhen Yu, Ancheng Wang, Jiajing Yin, Yongming Zhao, Jianrong Dong

**Affiliations:** 1 School of Nano-Tech and Nano-Bionics, University of Science and Technology of China, Hefei 230026, China; 2 Key Laboratory of Nano Devices and Applications, Suzhou Institute of Nano-Tech and Nano-Bionics (SINANO), Chinese Academy of Sciences, Suzhou 215123, China; 3 Dogain Laser Technology (Suzhou) Co., Ltd.; Suzhou 215000, China

**Keywords:** vertical-cavity surface-emitting lasers, surface grating, miniature atomic clock

## Abstract

Low threshold current and polarization-stabilized 795 nm vertical-cavity surface-emitting lasers (VCSELs) are fabricated by integrating a surface grating of high polarization selectivity and high reflectivity. The rigorous coupled-wave analysis method is used to design the surface grating. For the devices with a grating period of 500 nm, a grating depth of ~150 nm, and a diameter of the surface grating region of 5 μm, a threshold current of 0.4 mA and an orthogonal polarization suppression ratio (OPSR) of 19.56 dB are obtained. The emission wavelength of 795 nm of a single transverse mode VCSEL is achieved at a temperature of 85 °C under an injection current of 0.9 mA. In addition, experiments demonstrate that the threshold and output power also depended on the size of the grating region.

## 1. Introduction

Vertical cavity surface emitting lasers (VCSELs) are increasingly relevant for miniaturization and high integration applications, such as localized surface plasmon resonance (LSPR) sensors [1] and chip-scale atomic clocks (CSACs) [2], owing to a range of advantages including their compact package size, low threshold current, low power consumption, high modulation bandwidth, high-quality beam, and natural single longitudinal mode [3]. In all-optical coherent population trapping (CPT) atomic clocks, VCSELs are utilized as an attractive light source. However, to ensure the accuracy of the clock frequency and avoid introducing noise, the properties of the VCSEL must be stable over time, including its power, wavelength, and polarization [4]. To generate a pure circularly polarized light, the VCSEL must operate in a single polarization mode and pass through a quarter-wave plate (QWP) [4,5]. This is crucial in CPT atomic clocks, as the circular polarization is essential to excite atomic transitions from the ground state to the excited Zeeman sublevels. This process creates a CPT effect between the two ground state sublevels, resulting in the generation of a CPT clock signal [6]. However, the lack of an inherent polarization selection mechanism in VCSELs remains an issue due to their isotropic gain, cylindrical symmetrical resonator, and polarization-independent reflectivity. The conventional VCSELs based on GaAs and InP substrates produce linearly polarized light along $\left[011\right]$ or $\left[0\bar{1}1\right]$ at a certain injection current due to residual stress or electro-optic effect after fabrication [7], but the polarization of the VCSELs is not stable. Changing the driving current, temperature, or strain applied on the active region may lead to a polarization mode switching from one to another with a wavelength difference of ~100 pm between the polarization modes [8]. If the polarization of a VCSEL suddenly changes from the expected to the orthogonal direction, the resulting circular polarization will abruptly switch from right-circularly polarized to left-circularly polarized. This sudden change in polarization orientation will introduce noise into the clock signal, potentially affecting its accuracy. Therefore, a polarization-stabilized VCSEL with high polarization extinction ratio is critical for CPT atomic clocks to minimize noises.

Since the phenomenon of polarization switching of VCSELs was observed by Chang-Hasnain in 1991 [9], schemes based on different mechanisms have been proposed to control the polarization of VCSELs over the past few decades, e.g., polarization-dependent gain, asymmetric resonator, polarization-dependent reflector, and external optical feedback. Polarization-dependent gain can be introduced by applying anisotropic stress [10,11], growing epitaxial layer on (311B) substrates [12], and asymmetrically injecting current [13]. Rectangular [14], rhombus, and dumbbell mesas [15] are used to form asymmetric resonators introducing anisotropic gain or loss to achieve polarization control. Polarization-dependent mirror is usually realized by etching the surface grating with a certain period and depth in the cap layer of the VCSELs, producing an anisotropic reflectivity for the top distributed Bragg reflector (DBR) mirror. The polarization mode with higher reflectivity will preferentially lase and be the dominant polarization mode. Superior polarization stability has been achieved by integrating surface grating without significantly affecting the prime performance of the 895 nm VCSELs [16,17,18,19,20]. External optical feedback is also an effective way to control the polarization but requires a complex optical feedback structure and enlarges the device volume [21,22,23]. Among these polarization control technologies for VCSELs, the surface grating is the most promising one because of its advantages of monolithic integration, compatibility with standard VCSELs manufacturing processes, and high polarization stability. So far, most of the surface grating VCSELs have been focused on 850 nm or 894.6 nm VCSELs in data communication or cesium atomic clock, where the grating period, grating depth, and duty cycle typically are 600 nm, 60 nm, and 0.5, respectively [19,24,25]. However, 795 nm VCSELs have attracted more attention because of their applicability for rubidium atomic clocks, which provide a stable time and frequency reference for a variety of applications such as mobile and wired telecommunication infrastructure, broadcasting products, defense applications, calibration equipment and scientific instrumentation [26]. Investigations on 795 nm VCSELs have focused on improving their high-temperature performance, typically at 80 °C [27,28], or achieving stable single-mode operation at a wavelength of 795 nm with high output power at elevated temperatures [29,30]. However, there has been relatively less attention paid to the polarization control of 795 nm VCSELs. Without establishing a polarization selection mechanism in the device, a VCSEL will lase in a random polarization mode depending on the operating current and temperature [30]. In our previous work [31], the 795 nm in-phase surface grating VCSELs were designed and fabricated to stabilize the polarization, achieving a single mode and single polarization operation with an orthogonal polarization suppression ratio (OPSR) of 16.6 dB, which indicated a reasonably high polarization stability. However, that came at the cost of a 33.3% increase in the threshold current, which is caused by the lower reflectivity of the top distributed Bragg reflector (DBR) mirror after etching the grating. This paper proposes an anti-phase surface grating structure to minimize the increase in threshold current associated with the etching of the surface grating.

In this paper, the thickness of the cap layer is increased by λ/4 to a total thickness of 3λ/4 to meet the requirement of anti-phase gratings [18]. The experimental results show that a high OPSR and a low threshold current are realized in the devices with a larger surface grating region, while the output power degrades with the increasing size of the surface grating region. The fabricated surface grating 795 nm VCSELs show a low threshold current comparable with the device without gratings, and the OPSR reaches 19.56 dB.

## 2. Device Design and Fabrication

**Figure 1:**
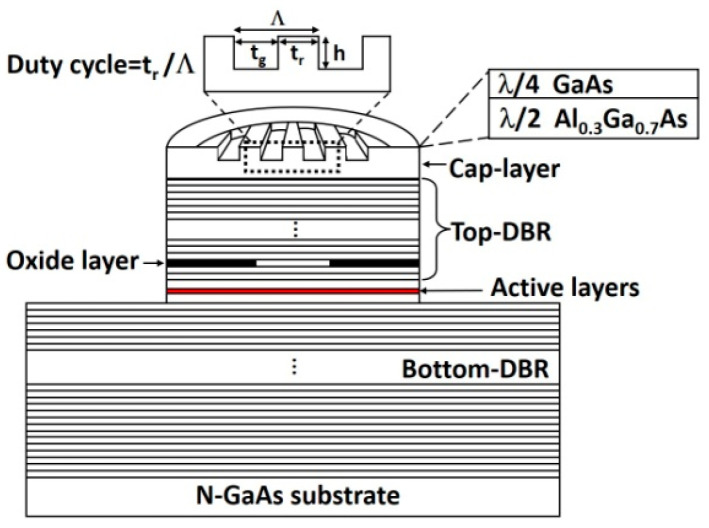
Schematic structure of a vertical-cavity surface-emitting lasers (VCSEL) with a surface grating period Λ, depth h, groove width $t_{g}$, and ridge width $t_{r}$, the duty cycle is defined by $t_{r}/\Lambda$.

**Figure 2:**
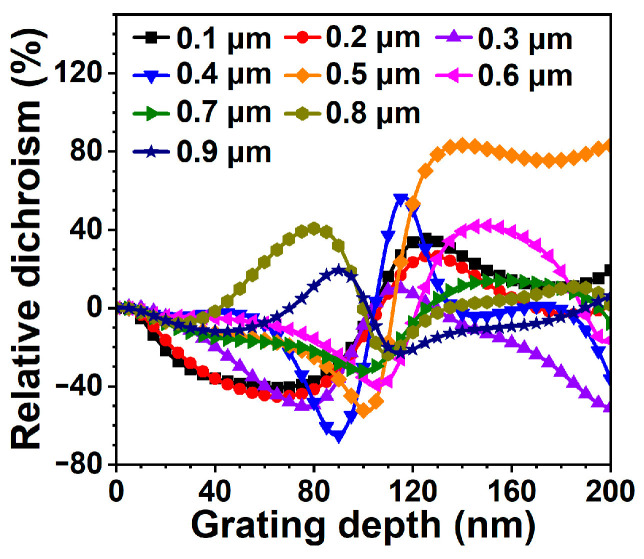
Dependence of relative dichroism (*RD*) on grating depth with the grating period as a parameter. The duty cycle is 0.5.

Figure 1 shows the schematic diagram of the device structure. The surface grating is etched in the cap layer on the top DBR mirror. Relative dichroism (*RD*) is usually used to estimate the intensity of polarization stability, which is defined by the following formula [16,24]

(1) $$RD=\left(1-\frac{G_{∥}}{G_{⊥}}\right)\times 100\%$$

where $G_{∥}$ and $G_{⊥}$ are the threshold gains of polarization modes parallel and orthogonal to the ridges of the grating, respectively. The output is dominated by the parallel polarization mode when *RD* > 0 due to the threshold gain of the parallel mode being smaller than that of the orthogonal polarization mode. For *RD* < 0 the dominant emission mode is orthogonal polarization. The threshold gain is defined by [32]

(2) $$g_{\mathrm{th}}=\alpha_{a}+\frac{1}{\Gamma_{r}d_{a}}\left[\alpha_{i}\left(L_{\mathrm{eff}}-d_{a}\right)+\ln\frac{1}{\sqrt{R_{t}R_{b}}}\right]\;\;$$

where $\alpha_{i}\;$ and $\alpha_{a}$ are intrinsic losses in the passive and active regions, respectively, $\Gamma_{r}$ is the optical confinement factor, $d_{a}$ is the total thickness of the active layers, $L_{\mathrm{eff}}$ is the effective cavity length and $R_{t}$ and $R_{b}$ are the reflectivity of the top and bottom DBR mirrors, respectively. The reflectivity of the top DBR mirror with the integrated surface grating for the two polarization modes are calculated by the rigorous coupled-wave analysis (RCWA) method [33]. The dependences of *RD* on grating periods and depths are calculated by Equations (1) and (2). The calculation results are plotted in Figure 2.

A similar dependence of |*RD*| on grating depth is observed for either positive or negative *RD*, i.e., the |*RD*| increases first and then decreases with the increasing grating depth. It can be seen from Figure 2 that the largest *RD* (87.21%) is obtained for the grating with a period of 500 nm and a depth of 140 nm. A high *RD* is maintained when the depth is increased up to 200 nm, allowing a larger tolerance for the grating depth, which is beneficial to the fabrication of the device and the improvement of the yield. In addition, the threshold gain of the device designed in our previous work, i.e., the grating period and depth are 200 nm and 70 nm, respectively, is about 2100 cm^−1^, but it is about 900 cm^−1^ in this work, which is a ~57% reduction compared with the prior work. Consequently, the 795 nm surface grating VCSELs designed and fabricated in this work show a higher OPSR and a lower threshold current.

**Figure 3:**
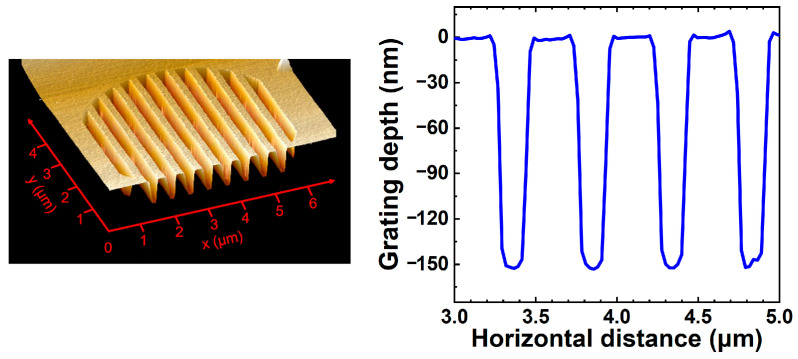
Atomic force microscopy (AFM) image and line-scan profile for a typical surface grating.

The epitaxial structure was grown on n-type (100) GaAs substrates by low-pressure metal-organic chemical vapor deposition (LP-MOCVD). The thickness of the cap layer composes of layers of λ/4-thick GaAs and λ/2-thick Al_0.3_Ga_0.7_As, meeting the condition of anti-phase [18]. The bottom and top DBR mirrors consist of 46.5 pairs Si-doped and 28 pairs C-doped Al_0.3_Ga_0.7_As/Al_0.9_Ga_0.1_As layers, respectively. A 20 nm-thick linearly composition-graded interface layer was inserted between the high and low Al-composition Al*_x_*Ga_1*−x*_As layers to reduce the series resistance. The active region with three 6.2 nm-thick Al_0.073_Ga_0.927_As quantum wells separated by 5 nm-thick Al_0.39_Ga_0.61_As barriers is sandwiched between two separate confinement layers. First, the surface gratings with a period of 500 nm, a depth of ~150 nm, and a duty cycle of 0.5 were fabricated using electron beam lithography (EBL) and inductively coupled plasma (ICP) etching techniques. Devices with surface grating periods of 200 nm, 700 nm, and 900 nm were also fabricated for comparison. To investigate how the size of the grating region affects the performances of VCSLEs, the diameters of the surface grating were varied from 2 μm to 5 μm. High-resolution positive photoresist, polymethyl methacrylate A2 resin with a molecular weight of 950 K, of approximately 130 nm was applied to the wafer by spinning coating for EBL. ICP etching was performed using Cl_2_ and BCl_3_ to form the surface grating at an etch rate of 10 nm/s. Atomic force microscopy (AFM) was used to analyze the profile of surface gratings. The AFM image of the fabricated surface grating is plotted in Figure 3. Then circle mesas with a diameter of 20 μm were etched to expose the layer of Al_0.98_Ga_0.02_As, which was then laterally oxidized to form an optical and electrical confinement aperture of ~4 μm by a selective wet oxidation process [34]. For planarization, the positive polyimide photoresist FB5410 from Fujifilm was coated on the P-side and cured for 1 h at 350 °C in a furnace. Subsequently, Ti/Pt/Au contact metals were deposited on the P-type topmost cap-layer by electron beam evaporation (EBE). Finally, the GaAs substrate was lapped to a thickness of ~150 μm, and the N-type contact metals of AuGe/Ni/Au were deposited using EBE, followed by rapid thermal annealing processing to form ohmic contacts. VCSELs without surface gratings were also fabricated as reference devices.

## 3. Results and Discussion

The light-current-voltage (L-I-V) characteristics of fabricated VCSEL chips were measured using a test system based on the LabVIEW platform, utilizing a source meter Keithley 2401 and a Newport power meter 1936-R. A Thorlabs LPNIRE100-B polarizer with a wavelength range of 600 to 1100 nm and a polarization extinction ratio of 400:1 was positioned between the VCSELs chip and the power sensor. The polarizer was oriented either perpendicular to or parallel to the grating ridges to measure the output powers of the two polarization directions. The optical powers were detected at normal incidence. Furthermore, the lasing wavelength of VCSEL chips were measured using an optical spectrum analyzer AQ6317. The thermal plate was utilized to control the chip temperature during the measurements.

**Figure 4:**
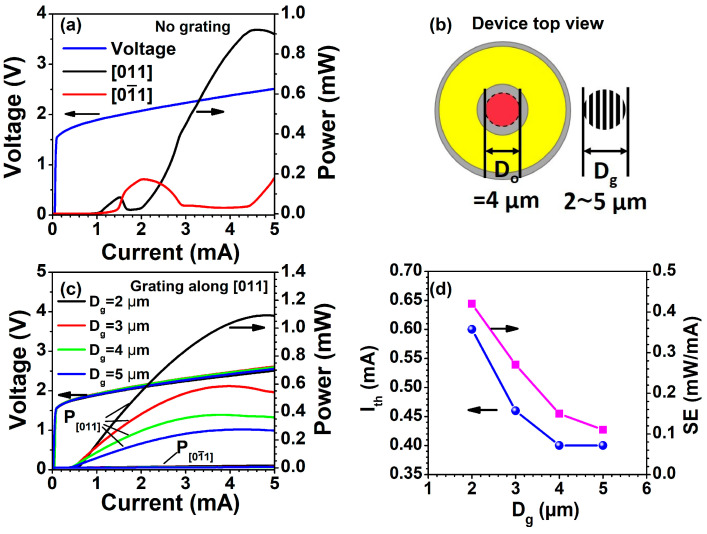
(**a**) Polarization-resolved light-current-voltage (L-I-V) characteristics of reference devices. (**b**) Schematic diagram of the surface grating (D_g_) and oxide aperture (D_o_). (**c**) Polarization-resolved L-I-V characteristics of VCSELs for different surface grating diameters with a grating period of 500 nm. (**d**) Dependence of threshold current (I_th_) and slope efficiency (SE) on D_g_ extracted from (**c**). The device was tested at room temperature (RT).

Figure 4a shows the room temperature (RT) polarization-resolved L-I-V characteristics curves of reference devices (without gratings). The polarization of the reference device switches at 1.5 mA and 2 mA. The phenomenon of polarization switching is often observed in conventional VCSELs due to the lack of a polarization control mechanism as mentioned earlier. In contrast, Figure 4c shows that the surface grating VCSELs produce a stable output polarization parallel to the grating ridge in the current range from threshold to thermal rollover for all the surface grating region diameters (D_g_) at RT. D_g_ and D_o_ are the diameters of the surface grating region and the oxide aperture, respectively, as illustrated in Figure 4b. From Figure 4c, it can be seen that the output power decreases from 1.1 mW to 0.23 mW as D_g_ increases from 2 μm to 5 μm at an injection current of 5 mA. The slope efficiency of the device decreases from 0.42 mW/mA to 0.11 mW/mA, and the threshold current decreases from 0.6 mA to 0.4 mA with the D_g_ increasing from 2 μm to 5 μm, as shown in Figure 4d. The grating period of 500 nm is larger than the critical value (234 nm for GaAs) of sub-wavelength grating, which introduces the diffraction loss [35], consequently, the output power and slope efficiency are lower compared with the previous results. The as-grown reflectivity of the top DBR mirror calculated by the transfer matrix method [36] is ~94%, lower than the normal device (99.4%) due to the addition of λ/4 thickness to the top layer, but it can be raised to more than 99% by etching the grating, depending on the grating period and depth. Therefore, when D_g_ < D_o_, the region of the emission window without grating (D_o_-D_g_) has a lower reflectivity (higher diffraction loss) compared to the grating region, thus the threshold current decreases with the increasing D_g_. When D_g_ ≥ D_o_, the oxide aperture is completely covered by the grating region, a constant high reflectivity is achieved, so the threshold current does not decrease further with D_g_ exceeding 4 μm as shown in Figure 4d, while the output power keeps decreasing because the diffraction loss increases with D_g_, as shown in Figure 4c.

**Figure 5:**
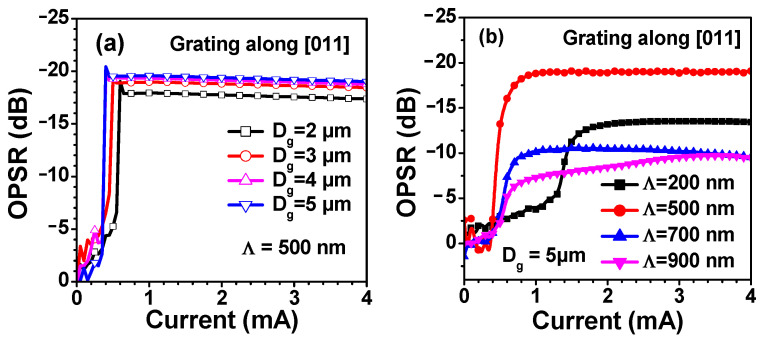
(**a**) OPSR of VCSELs for different surface grating areas with a period of Λ = 500 nm. (**b**) OPSR of VCSELs with different grating periods at D_g_ = 5 μm.

The OPSR is defined by [24]

(3) $$\mathrm{OPSR}=10\times \log\left(\frac{P_{⊥}}{P_{∥}}\right)\;\;$$

where $P_{⊥}$ and $P_{∥}$ are the optical power of the polarization mode orthogonal and parallel to the grating ridges, respectively. The size of the surface grating region also affects the OPSR, as shown in Figure 5a. The OPSR slightly increases with the increasing D_g_. When D_g_ = 5 μm, the OPSR of the device reaches 19.56 dB once the injection current is above the threshold current. OPSRs of the devices with different grating periods are shown in Figure 5b. It can be seen that the device with a 500 nm grating shows the best polarization control, which is consistent with the calculated results shown in Figure 2.

**Figure 6:**
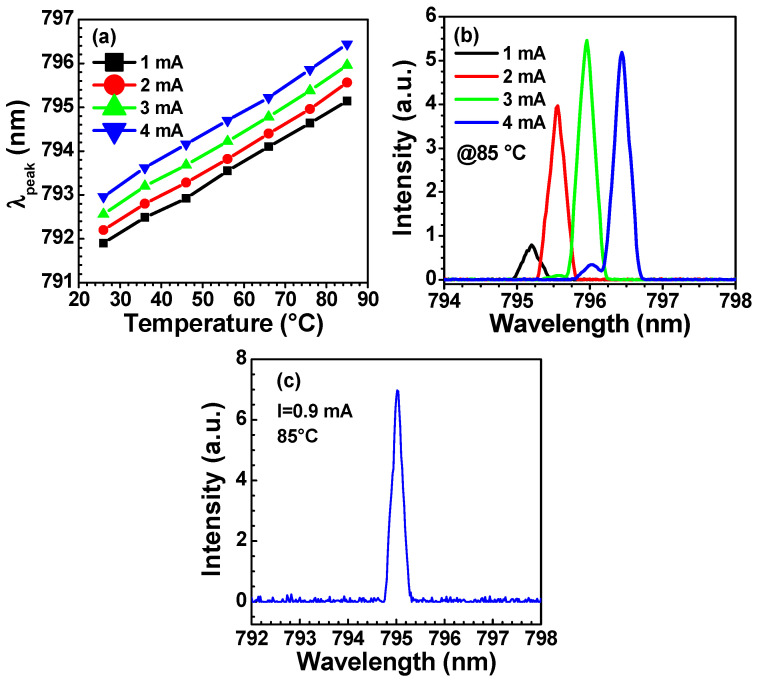
(**a**) Temperature dependence of the peak wavelength (λ_peak_) at different injection currents. (**b**) Emission spectrum of the VCSEL at 85 °C and different injection currents. (**c**) Emission spectrum of the VCSEL at 85 °C and an injection current of 0.9 mA. The device has a surface grating period of 500 nm, a depth of 150 nm, and a D_g_ = 5 μm.

The lasing peak wavelength (λ_peak_) of a VCSEL changes with temperature and injection current. Figure 6a shows the temperature dependence of the peak wavelength at different injection currents. For an injection current of 1 mA, the emission wavelength is 791.8 nm at 26 °C, and its red-shift with increasing temperature reaches 795.14 nm at 85 °C. The fitted temperature coefficient is 0.055 nm/K and 0.058 nm/K for injection currents of 1 mA and 4 mA, respectively. This temperature coefficient difference indicates a higher wavelength red-shift rate at a higher injection current level. Figure 6b shows the emission spectra of a 500 nm-period surface grating VCSEL at 85 °C at different injection currents. It can be seen that the device remains a single transverse mode operation below 3 mA. The peak wavelength shifts at a rate of 0.38 nm/mA in the current range of 1 to 3 mA. Taking into account the temperature and injection current dependence of VCSEL lasing wavelength, the VCSEL emission wavelength at 85 °C can be tuned to 795 nm by adjusting the injection current to 0.9 mA, as shown in Figure 6c, which corresponds to the D1 line of the rubidium atomic clock [37].

**Figure 7:**
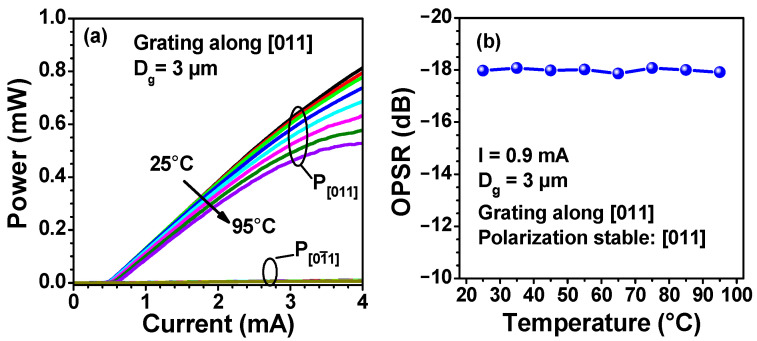
Light-current (L-I) characteristics (**a**) and OPSR at an injection current of 0.9 mA (**b**) of a surface grating VCSEL at different temperatures. The period and depth of the surface grating are 500 nm and ~150 nm, respectively.

Figure 7a shows the light-current (L-I) curves of inverse surface grating VCSELs measured under different temperatures. The slope efficiency decreases from 0.28 mW/mA to 0.19 mW/mA and the threshold current increases from 0.5 mA to 0.64 mA as the temperature increases from 25 °C to 95 °C. The dominant polarization remains in [011] direction and the OPSR at an injection current of 1 mA is around 18 dB in the temperature range investigated, as shown in Figure 7b, indicating a stable polarization even at a high temperature.

**Figure 8:**
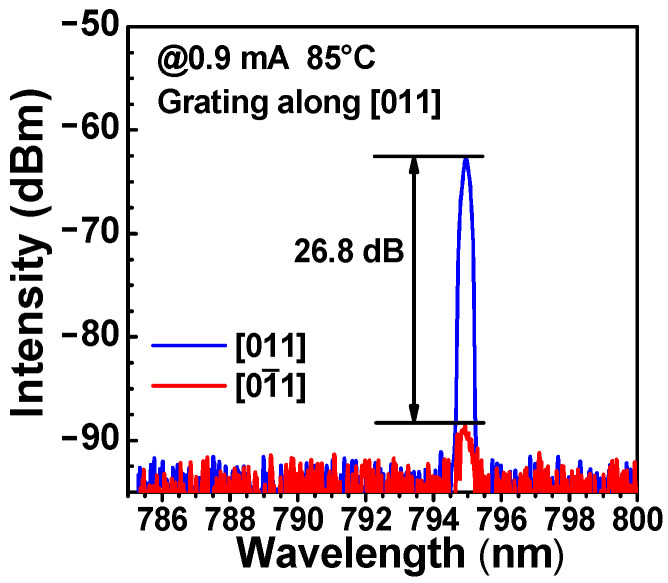
Polarization-resolved spectra of a 795 nm VCSEL at an injection current of 0.9 mA and 85 °C.

Figure 8 shows the polarization-resolved spectra of the device at a temperature of 85 °C and an injection current of 0.9 mA. It can be seen that the peak-to-peak OPSR reaches 26.8 dB. The fabricated grating VCSELs demonstrate the characteristics of low power consumption, single transverse mode, and high polarization stability, showing the promising potential of being applied to chip-scale rubidium atomic clock systems.

## 4. Conclusions

In conclusion, based on the calculation of the polarization characteristics of the inverse surface grating VCSELs by RCWA, the optimized grating parameters of 795 nm VCSELs are designed. Subsequently, surface grating VCSELs are fabricated and characterized. The results demonstrated that an OPSR of 19.56 dB is achieved for VCSELs with a grating period, depth, and duty cycle of 500 nm, 150 nm, and 0.5, respectively. Experiments found that the slope efficiency, output power, threshold current, and OPSR of the surface grating VCSELs were affected by the size of the surface grating region. The slope efficiency and threshold current decrease with the increasing D_g_, while the threshold current remains constant for D_g_ ≥ D_o_. A larger grating area would be beneficiary to the improvement of the OPSR of the devices. The grating size dependence of the device performance provides another parameter for VCSEL design.

## Data Availability

Data available on request due to restrictions e.g., privacy or ethical.
